# Capturing One of the Human Gut Microbiome’s Most Wanted: Reconstructing the Genome of a Novel Butyrate-Producing, Clostridial Scavenger from Metagenomic Sequence Data

**DOI:** 10.3389/fmicb.2016.00783

**Published:** 2016-05-26

**Authors:** Patricio Jeraldo, Alvaro Hernandez, Henrik B. Nielsen, Xianfeng Chen, Bryan A. White, Nigel Goldenfeld, Heidi Nelson, David Alhquist, Lisa Boardman, Nicholas Chia

**Affiliations:** ^1^Microbiome Program, Center for Individualized Medicine, Mayo Clinic, RochesterMN, USA; ^2^Department of Surgery, Mayo Clinic, RochesterMN, USA; ^3^Carl R. Woese Institute for Genomic Biology, University of Illinois at Urbana-Champaign, UrbanaIL, USA; ^4^Roy J. Carver Biotechnology Center, University of Illinois at Urbana-Champaign, UrbanaIL, USA; ^5^Center for Biological Sequence Analysis, Department of Systems Biology, Technical University of DenmarkKongens Lyngby, Denmark; ^6^Department of Health Sciences Research, Mayo Clinic, RochesterMN, USA; ^7^Department of Animal Sciences, University of Illinois at Urbana-Champaign, UrbanaIL, USA; ^8^Department of Physics, University of Illinois at Urbana-Champaign, UrbanaIL, USA; ^9^Division of Gastroenterology and Hepatology, Mayo Clinic, RochesterMN, USA; ^10^Department of Physiology and Biomedical Engineering, Mayo Clinic, RochesterMN, USA

**Keywords:** microbiome, metagenomics, *Butyricicoccus*, binning, genome assembly

## Abstract

The role of the microbiome in health and disease is attracting great attention, yet we still know little about some of the most prevalent microorganisms inside our bodies. Several years ago, Human Microbiome Project (HMP) researchers generated a list of “most wanted” taxa: bacteria both prevalent among healthy volunteers and distantly related to any sequenced organisms. Unfortunately, the challenge of assembling high-quality genomes from a tangle of metagenomic reads has slowed progress in learning about these uncultured bacteria. Here, we describe how recent advances in sequencing and analysis allowed us to assemble “most wanted” genomes from metagenomic data collected from four stool samples. Using a combination of both *de novo* and guided assembly methods, we assembled and binned over 100 genomes from an initial data set of over 1,300 Gbp. One of these genome bins, which met HMP’s criteria for a “most wanted” taxa, contained three essentially complete genomes belonging to a previously uncultivated species. This species is most closely related to *Eubacterium desmolans* and the clostridial cluster *IV/Clostridium leptum* subgroup species *Butyricicoccus pullicaecorum* (71–76% average nucleotide identity). Gene function analysis indicates that the species is an obligate anaerobe, forms spores, and produces the anti-inflammatory short-chain fatty acids acetate and butyrate. It also appears to take up metabolically costly molecules such as cobalamin, methionine, and branch-chained amino acids from the environment, and to lack virulence genes. Thus, the evidence is consistent with a secondary degrader that occupies a host-dependent, nutrient-scavenging niche within the gut; its ability to produce butyrate, which is thought to play an anti-inflammatory role, makes it intriguing for the study of diseases such as colon cancer and inflammatory bowel disease. In conclusion, we have assembled essentially complete genomes from stool metagenomic data, yielding valuable information about uncultured organisms’ metabolic and ecologic niches, factors that may be required to successfully culture these bacteria, and their role in maintaining health and causing disease.

## Introduction

The vast majorities of bacteria within and around us have not been successfully cultured (Torsvik et al., 1990; Fierer et al., 2008), and are thus difficult to identify, much less study. The field of metagenomics seeks to address this problem by directly accessing the genomes of uncultured organisms and analyzing their contents (Handelsman et al., 1998; Handelsman, 2004). On the one hand, recent sequencing and analytical advances and efforts such as the Human Microbiome Project (HMP) (Gevers et al., 2012) and the Metagenomics of the Human Intestinal Tract (MetaHIT) project (Qin et al., 2010) have allowed us to learn more about our “microbial dark matter” (Lok, 2015). On the other hand, some of the most common microbial residents of our bodies remain elusive, leading Fodor et al. (2012) to compose a “most wanted” List of HMP taxa. Four years ago, they issued a challenge to the research community to sequence the genomes of these “most wanted” organisms, which were prioritized based on their frequency in healthy HMP volunteers and their distance to already sequenced organisms. Simply by characterizing these organisms’ gene repertoires, we can glean key information about their functional niches in the body: what compounds they synthesize, how they use the metabolites present in their environment, the mechanisms they use to interact with or invade our cells, and whether they are resistant to antibiotics.

Although there is consensus that analyzing the genomes of these taxa is essential to understanding the role of the microbiome in maintaining health, the challenges associated with assembling specific genomes from the complex morass of metagenomic data have slowed progress toward this goal. Recently, MetaHIT researchers made headway by identifying sets of coabundant gene groups (CAGs) in metagenomic data from multiple samples, and assigning these CAGs to individual microbial species (Nielsen et al., 2014). The next logical goal is to use a similar approach to assemble whole genomes from metagenomic data. Although initially it was only possible for less complex microbial communities, assembling genomes directly from the environment has gained traction thanks to the improved approaches and software platforms, such as differential coverage and k-mer–based binning methods, which open potential avenues for their increased use in the study of ecosystems such as the gut microbiome (Albertsen et al., 2013; Alneberg et al., 2013; Sharon and Banfield, 2013; Eren et al., 2015).

We harnessed these recent advances in sequencing and metagenomic analysis to analyze the microbes present in four stool samples and to obtain three essentially complete genomes belonging to an uncharacterized species that met the HMP’s “most wanted” criteria. Based on the metabolic profiles obtained from these genomes, we predict that this species is a scavenger dependent on metabolites produced by other gut bacteria, that successful culture may depend on supplementation with methionine and cobalamin, and also that the bacterium produces the anti-inflammatory small-chain fatty acids (SCFAs) acetate and butyrate, making it a potential candidate for probiotic therapy. Thus, we show that, using readily accessible methods, it is possible to assemble the genomes of novel species from metagenomic data and to gain insight into their ecological niches, nutrient requirements, and potential roles in maintaining health.

## Materials and Methods

### Subject Consent and Sampling

Four stool samples were collected prior to colonoscopy as part of a broader ongoing study of patients at the Mayo Clinic. Two (A254 and K4410) were from subjects diagnosed with colon cancer, and two (N15 and N54) were from subjects with a negative colonoscopy. This study was reviewed and approved by the Institutional Review Board at Mayo Clinic under protocol numbers 10-004833 and 10-006009, and written informed consent was obtained from all participants.

### DNA Extraction

Genomic DNA (gDNA) was extracted from the stool samples using the PowerSoil DNA isolation Kit (MO BIO Laboratories, Carlsbad, CA, USA). Briefly, for each sample, we created 24 aliquots, each containing approximately 150–200 mg stool. These aliquots were then processed together in a single batch. After adding 60 μl buffer C1 to each aliquot, the samples were homogenized in PowerBead tubes on the FastPrep-24 (MP Biomedicals, Santa Ana, CA, USA; settings: *S* = 4, *T* = 40 s). Next, the supernatant was processed following the manufacturer’s protocol with a few modifications: 70 μl buffer C6 was loaded to the spin filter, and the product was loaded for a second elution to increase its concentration. All eluted products from the same stool sample were then pooled and quantified with the Qubit fluorometer (Invitrogen, Carlsbad, CA, USA). In the case of insufficient yield, more stool aliquots were processed, to yield at least 1 μg gDNA from each sample.

### Construction of Shotgun and Mate-Pair Libraries

To minimize misassemblies due to repeat regions, we created both shotgun and mate-pair libraries, which allowed us to sequence genomic regions separated by short and long distances. The multiple library fragment size ranges allowed us to reach past repeat regions, and multiple mate-pair size selections granted us access to extra sources of validation for scaffolding. The libraries were constructed at the Roy J. Carver Biotechnology Center, University of Illinois at Urbana-Champaign (UIUC), which also carried out sequencing. Shotgun gDNA libraries were constructed with the Library Preparation Kit from Kapa Biosystems (Wilmington, MA, USA) from 1 μg of DNA sonicated with a Covaris M220 (Woburn, MA, USA). These libraries were loaded onto a 2% agarose gel, and fragments 400–600 bp in length were selected. Final libraries were run on high-sensitivity DNA chips (Agilent, Santa Clara, CA, USA) to determine the average fragment size and to confirm the presence of DNA in the expected size range. The libraries were also quantitated by qPCR on a BioRad CFX Connect Real-Time System (Hercules, CA, USA).

To generate reads with longer insert sizes, mate-pair libraries were prepared with the Nextera Mate-Pair Sample Preparation Kit (Illumina; San Diego, CA, USA). Briefly, 10 μg high-quality gDNA was subjected to two tagmentation reactions and run on a 0.6% Mb agarose gel. Genomic fragments 4–6 kb, 8–12 kb, and 15–20 kb in size were selected, purified on an EluTrap (GE Healthcare Life Sciences; Piscataway, NJ, USA), and circularized. The circles were sonicated with a Covaris M220 (Covaris, Woburn, MA, USA) and enriched for fragments containing the biotinylated circularization adapter. Enriched fragments were then end-repaired, A-tailed, adaptered, and PCR amplified with the TruSeq DNA Sample Prep kit (Illumina). Final libraries checked for size and quantitated as described above.

### Sequencing on an Illumina HiSeq2500

The libraries were sequenced on an Illumina HiSeq2500. DNA fragments were sequenced for 151 cycles from each end using TruSeq Rapid SBS v1 sequencing kits. Then, the raw.bcl files were converted into demultiplexed compressed fastq files using bcl2fastq v1.8.2 Conversion Software (Illumina).

### Preprocessing of Sequenced Reads

#### Removal of Human Host Reads

First, we removed raw reads identified as human sequences. To do so, we ran Kraken v0.10.4-beta (Wood and Salzberg, 2014) trained with the *Homo sapiens* reference from the 1000 Genomes Project, bacterial and archaeal genomes from NCBI’s “Bacteria” collection, and viral genomes from NCBI’s “Viruses” collection. This step was repeated as necessary during read processing, as described below.

#### Quality Trimming and Adapter Removal

Next, we removed sequencing adapters and trimmed low-quality bases from the reads using Trimmomatic v0.32 (Bolger et al., 2014) and the following settings: ILLUMINACLIP:adapters.fasta:2:30:10 LEADING:3 TRAILING:3 MAXINFO:100:0.1 MINLEN:60. The “adapters.fasta” file, which contains a description of the Illumina TruSeq3 and Nextera adapters, is distributed with the Trimmomatic package. Singleton, orphan reads (i.e., reads that lost their mate during the filtering step) were retained as single reads.

#### Error Correction

Error correcting reads increases assembly quality by removing k-mers when there is statistical or quality evidence they come from sequencing errors, and this step is usually included in single-genome assemblers such as SPAdes, but not to our knowledge for metagenomic assemblers. We performed basic error correction of the reads using the error correction step of the SGA assembler (Simpson and Durbin, 2012). For preprocessing, the following command was used: sga preprocess –dust –no-primer-check -m 60; for correction, we used the command sga correct -k 55 –discard –learn.

#### Digital Read Coverage Normalization

Read coverage normalization is a step needed when the assembly process would require computer memory resources that exceed the available capacity, at the expense of discarding some of the read data, and deep metagenomes like the one in this study can easily exceed this capacity. Read coverage was independently digitally normalized with khmer v1.3 (Crusoe et al., 2015). For each of a sample’s libraries, we performed a single-pass digital normalization to 60X using the command normalize-by-median.py -k 20 -N 4 -x 3e10 -C 60, with a false-positive rate less then 0.001 (we increased the value of -x if the false-positive rate was larger than 0.001). Then we performed abundance filtering using the command filter-abund.py -V.

### Metagenomic Assembly

#### Removal of Host Reads and Assembly

We performed a second pass of Kraken on the normalized reads to remove human host reads.

We then assembled each sample separately, using all available libraries, with Ray-Meta version 2.3.1 (Boisvert et al., 2012), using a k-mer value of 39. The annotation step of the program was skipped. Finally, we ran Kraken a third time to discard newly identified human host reads. By using the normalized read libraries, we ensured RAM usage during assembly was within the capacity of our systems.

#### *De Novo* Binning and Reassembly

To perform *de novo* binning, we followed the steps outlined by Albertsen et al. (2013). Briefly, we annotated the assembled contigs using per-sample coverage information, consensus taxonomy, GC content, and tetranucleotide spectrum information (for information on how we calculated GC content and tetranucleotide spectrum information, see below). We then used the differential coverage information, taxonomy, and GC content to manually bin clusters of contigs putatively belonging to the same organism; tetranucleotide spectrum values were used to remove contigs dissimilar to the bulk of other contigs in a bin.

##### Coverage annotation of assembled contigs

We extracted contigs larger than or equal to 1,000 bp and mapped error-corrected, un-normalized reads from all samples onto the contigs using BWA-mem version 0.7.6a (Li and Durbin, 2009). We then calculated per-sample coverage using SAMtools version 0.1.19-44428cd (Li et al., 2009) and bedtools version 2.16.2 (Quinlan, 2014).

##### Taxonomy annotation of assembled contigs

For contigs longer than 1,000 bp, we predicted genes using MetaProdigal version 2.60 (Hyatt et al., 2012). We queried the peptide sequences of the predicted genes against the RefSeq protein database (performed on October 29, 2013) using USEARCH version 6.0.307 (Edgar, 2010) in uBlast mode, with an e-value of 1e-5, maxhits set to 5, and accel at 0.9 (command: usearch6.0.307_i86linux64 -ublast queries.faa -db refseq.udb -evalue 1e-5 -maxhits 5 -accel 0.9 -blast6out output.txt). We then parsed the USEARCH output to determine the taxonomy lineage of the lowest common ancestor (LCA) for each predicted gene using blast2lca (Altschul et al., 1990; Pignatelli, 2014) against the NCBI taxonomy (obtained on October 29, 2013) and summarized the LCA for a contig using the voting scheme implemented in the script hmm.majority.vote.pl, used in Albertsen et al. (2013).

##### GC and tetranucleotide spectrum annotation

We calculated per-contig GC content and the canonical (strand-independent) tetranucleotide (4-mer) spectrum using custom scripts. Instead of saving the raw 4-mer counts per contig or the frequency of each 4-mer per contig, we saved the *z*-score of each 4-mer per contig.

##### Manual identification of bins

As outlined in Albertsen et al. (2013), we created differential coverage plots annotated with the taxonomy and sequence data described above. To reduce clutter, we split the data by phylum (Firmicutes, Bacteroidetes, Actinobacteria, Proteobacteria, and “other phyla”) and then examined the differential coverage plots. In the case of the Firmicutes and Bacteroidetes, we split the data even further, to the order or family level when possible. Once we identified a set of contigs, we used the corresponding tetranucleotide spectra to perform principal components analysis (PCA), plotting the contigs against the first and second dimensions. We selected the contigs that clustered together, with the presumption that outliers did not belong in the bins.

##### Mapping and reassembly

We mapped error-corrected, un-normalized reads from all eight libraries (one shotgun and one mate-pair library for each sample) onto the preliminary bins using BWA-mem. We have found that combining velvet and SPAdes produces more complete bin assemblies. Thus, we performed a first-pass assembly using velvet version 1.2.10 (Zerbino and Birney, 2008) with k-mer values from *k* = 19 to *k* = 99, retaining contigs longer than 1,000 bp. Then, we performed a second-pass assembly using SPAdes version 3.1.0 (Bankevich et al., 2012), passing the velvet contigs from the best assembly (containing the longest contig) to SPAdes using the “untrusted-contig” parameter. Using Prodigal and HMMER version 3.0 (Eddy, 2011), we identified each bin’s essential genes using the hidden Markov models (HMM) used for essentiality tests in Albertsen et al. (2013), and we declared a bin “essentially complete” if we found 100 or more unique genes from this HMM set, which contains profiles for 111 genes. We also searched for 16S rRNA reads using RNAmmer (Lagesen et al., 2007). Finally, if 16S rRNA and *rpoB* sequences were present among the essential genes, we used them to taxonomically identify the bin and also as potential markers for contaminant contigs. At this point, the bins were ready for further contamination and completeness checks, outlined below.

#### Supervised Binning and Reassembly

##### Genome references

First, we selected references for guided reconstruction of genomes: 741 metagenomic species (MGSs) identified as part of a large collection of metagenomic libraries of the human intestinal tract (Nielsen et al., 2014). The MGSs are groups of at least 700 genes whose abundance across samples is highly correlated; they served as seeds for the progressive assembly of genomes.

##### Read mapping and rRNA removal

Next, we ensured that the highly conserved rRNA operon, which receives high coverage, did not interfere with the assembly process. The reason is that rRNA is highly conserved across species, and when mapping reads onto the MGS bins there were regions of extremely high coverage which may result in chimeric contigs. To avoid this, we decided to discard these reads for this assembly step. To do so, we mapped the un-normalized reads described earlier onto the genes of each MGS bin using BWA, discarding unmapped read pairs. We then mapped the resulting reads onto the SILVA database version 119 (Quast et al., 2013), which contains 16S and 23S rRNA reads, and removed reads that aligned to the SILVA reference.

##### Bin assembly and cleanup

We assembled the bin reads using SPAdes with k-mer values of “21,33,55,77,” “21,33,55,77,99,” and “21,33,55,77,99,127,” retaining the assembly with the longest assembled contig for further processing. We then removed contigs shorter than 1,000 bp and with reported coverage less than 2X. Putative contaminant sequences (i.e., those not belonging to the bins) were removed using BioBloom tools version 2.0.6 (Chu et al., 2014), trained with either the original MGS gene bin or with the corresponding published assembly, if available. We continued removing putative contaminants using CheckM version 0.9.7 (Parks et al., 2015) by calculating the bin distribution of GC content, coding density, and tetranucleotide spectrum, and then removing outliers - -contigs falling outside of the 95% Confidence Intervals for these distributions- - through the “checkm outliers” command. After this step, we discarded any bin with less than 100,000 bp assembled.

##### Reiteration of the process

In order to continue improving the assemblies and removing low quality contigs, the resulting bins were used to reiterate the steps above, starting with mapping. Briefly, we mapped un-normalized reads onto the bin obtained from the previous iteration, mapped against SILVA 119 to discard rRNA, reassembled with SPAdes, filtered out short and low-coverage contigs, removed putative contaminants using BioBloom tools trained with the bin obtained from the previous iteration, and removed outliers using CheckM. After repeating this process three times, we found little additional changes on both contigs added and low quality assemblies removed and therefore we terminate the iterative clean-up process.

##### Extension of assembled contigs

To complete the assembly process, we use a specialized assembler that extends the contigs from their tips using the unassembled reads. For some of the contigs, this has the effect of re-introducing the rRNA operons, and by requiring high mapping identities to the ends of the contigs, the process will likely not introduce misassemblies. To extend the contigs obtained from the binning process, we used the PRICE genome assembler version 1.2 (Ruby et al., 2013), which maps reads onto the ends of contigs and iteratively extends them. We used the normalized paired-end reads and ran the program for 10 cycles, with parameters -fpp reads_R1.fastq reads_R2.fastq 500 95 -icf my_bin.fasta 1 1 5 -nc 10 -dbmax 72 -mol 30 -tol 20 -mpi 90 -target 90 0, extending input contigs but not creating new ones. We discarded bins in which the extension process was not finished. In addition, when PRICE extends contigs with highly repetitive regions, it appears the memory usage increases until all available memory is saturated. Consequently, the bins containing a high number of repeat regions may not be present at all. For this reason, we also discarded bins that used excessive memory.

##### Completeness check and final contamination removal

Using CheckM, we estimated each bin’s completeness and contamination level, and we retained bins with completeness greater than 70% and contamination less than 5% for further processing. To assign taxons, we ran Prodigal to predict genes, used USEARCH to find matches in RefSeq in uBlast mode, and used blast2lca to estimate an LCA taxonomic assignment for the bins. We then ran ProDeGe version 2.2 (Tennessen et al., 2015) on each bin to remove additional putative contaminants, passing the estimated taxonomy assignment as a parameter.

### Identification of the “Most Wanted” Taxa in Stool Samples

Following the same criteria as set forth by Fodor et al. (2012), we began by determining which organisms were present in the Midwest Reference Panel (MWRP) (Chen et al., 2016), a recently described gut microbiome sample representative of the volunteers who gave stool samples for this project. Operational taxonomic units (OTUs) present in the MWRP were searched against 16S genes extracted from 12,724 human-associated genomes from the Genomes Online Database Human (GOLD-Human) version 5 (as of September 28, 2015) (Reddy et al., 2015) and also from HMP strains (Fodor et al., 2012), and sequence identity was calculated using PyNAST (Caporaso et al., 2010). In addition, the prevalence of OTUs was calculated by counting the number of MWRP samples in which each OTU was present. This allowed us to separate high-priority, medium priority, and low-priority genomes based on genetic novelty and prevalence in the human gut. As a simple example, a genome that has unique characteristics and is present in all of us would have the highest priority. As per the Fodor et al. (2012) criteria, “most-wanted” OTUs were those with 90% or less similarity to a sequenced genome and a prevalence greater than or equal to 20% in the MWRP cohort. Medium priority genomes were those between 90 and 97% similarity to a sequenced genome and prevalence greater than or equal to 20% in the MWRP cohort. The remaining genomes were low priority.

**FIGURE 1:**
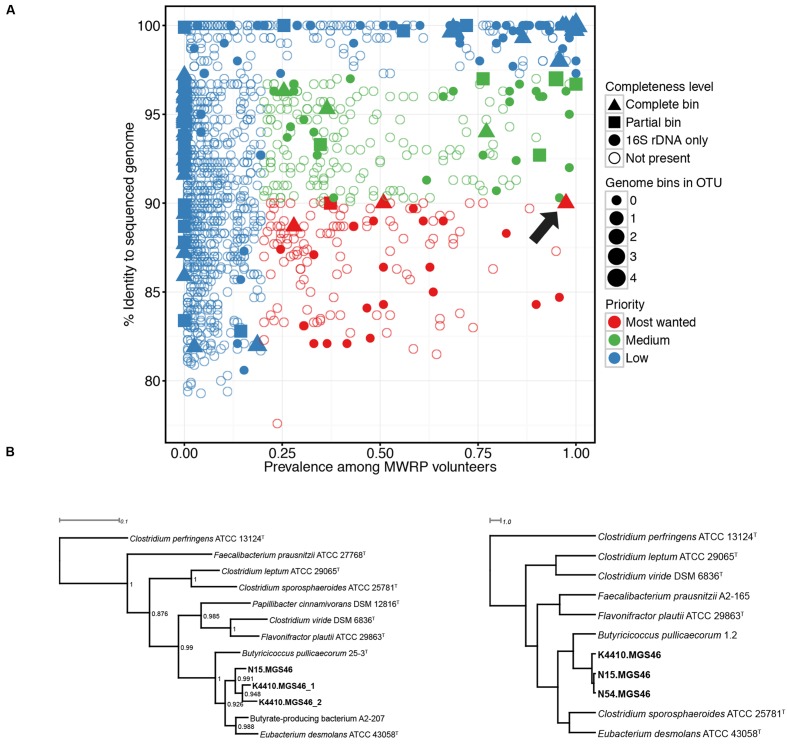
**(A)** Numerous “most wanted” taxa were present in samples from the Midwestern Reference Panel (MWRP), and one essentially complete “most wanted” bin was present in metagenomic data gathered from four stool samples. 16S amplicons were used to establish the prevalence of various taxa among the MWRP volunteers and the distance of those taxa from already sequenced organisms, as determined by matches to the Genomes Online Database Human-associated genomes (GOLD-Human) or the Human Microbiome Project Strains. The size of each bin indicates the number of hits to that OTU in the MWRP. As in Fodor et al. (2012), prevalent taxa (>20%) that were not closely related to already sequenced organisms (<90%) were assigned highest priority. Colors represent “most wanted” status: red, high priority; green, medium priority; blue, low priority. Filled shapes indicate the binned genomes in our stool metagenomic data set that matched operational taxonomic units (OTUs) from the MWRP with 97% or higher similarity. Shapes indicate the completion status of the binned genome in our metagenomic data set: triangle, complete sequence data; square, partial sequence data; circle, 16S-only sequence data. The arrow points to the *Butyricicoccus* sp. B MC bin, which we selected for further study. **(B)** Phylogenies show that the closest relatives of the three assembled genomes (*Butyricicoccus* sp. B MC N15, -N54, and -K4410) within the essentially complete “most wanted” bin are clostridial cluster IV/*Clostridium leptum* subgroup strains. Left: 16S rRNA phylogeny of the assembled genomes together with type strains from species of the clostridial IV/*Clostridium leptum* subgroup. *Clostridium perfringens* is an outgroup, and bootstrap values are shown for each node. *Note*: N54 is missing because its 16S sequence is not available. Right: Tetranucleotide spectrum–based whole-genome phylogeny of the three reconstructed genomes together with genomes from the clostridial cluster IV/*C. leptum* subgroup type species. Both phylogenies identify the nearest neighbors as *Butyricicoccus pullicaecorum* and *Eubacterium desmolans*.

Next, we determined which high-priority MWRP OTUs were present in the metagenomic data from the four stool samples we collected. To do this, 16S reads extracted from the assembly were clustered against the MWRP OTUs, and reads were deemed a hit if there was 97% or higher sequence similarity. Genomic bins with no identity to an OTU in the MWRP were defined as zero prevalence. We also used completeness as a criterion to decide which bin to characterize further, with essentially complete bins (as defined above) receiving higher priority. Based on “most-wanted” and completion status, we chose three genomes from bin MGS46 for additional study. They were essentially complete, 90.0% similar to the closest publicly available genome, and present in 97.5% of MWRP volunteers (**Figure 1**, **Supplementary Table S1**).

### Annotation of Genomes

Genomes were annotated using RASTtk (Brettin et al., 2015) through PATRIC (Snyder et al., 2007). Functional characterization was also performed using PATRIC.

### Phylogeny and Functional Comparison

A 16S rRNA phylogeny and two types of whole-genome phylogeny were constructed to determine where the three selected genomes fell on the tree of life. These different types of phylogeny provide complementary information. 16S rRNA phylogenies allow deep, long-range reconstructions of evolutionary relationships but lack resolution below the species level. By contrast, whole-genome clustering using BLAST-based average nucleotide identity (ANIb) provides strain-level resolution and correlates well with DNA-DNA hybridization (DDH) cutoffs for species (70% DDH is equivalent to 95% ANIb). ANIb phylogenies become unreliable for identities lower than 80%, however, due to low levels of the overlap necessary to calculate identity (Rosselló-Móra and Amann, 2015). An alternate method bridges the gap between ANIb and 16S rRNA phylogenies by using the whole genome to construct a tetranucleotide spectrum, which is used to calculate distances with reliability that extends beyond the species level (Teeling et al., 2004).

The 16S phylogeny was constructed by aligning the 16S sequences of the reference and assembled organisms using Infernal, with the bacterial covariance model from RDP (Cole et al., 2009). An approximate maximum likelihood phylogeny was computed using FastTree (Price et al., 2010) with the parameters -nt -gtr -gamma -slownni -spr 4 -mlacc 2. The ANIb phylogeny was computed by using JSpecies (Richter and Rosselló-Móra, 2009) and then performing hierarchical clustering using the apcluster package. The tetranucleotide whole-genome phylogeny was constructed by computing the canonical tetranucleotide spectra of all genomes (Teeling et al., 2004), transforming these data into *z*-scores and calculating the Euclidian distance, and then performing exemplar-based agglomerative clustering (a hierarchical clustering method) using the R package apcluster (Frey and Dueck, 2007; Bodenhofer et al., 2011).

A pangenome analysis of the three assembled genomes and their two nearest neighbors was performed using the GView Server (Petkau et al., 2010).

Usage of the clusters of orthologous groups (COGs/NOGs) (Huerta-Cepas et al., 2015) was computed by mapping gene annotation onto COG categories and then normalizing by the total number of hits per genome.

A functional hierarchy was calculated by determining the FIGfams functional families (Meyer et al., 2009) present in each genome and then calculating the Jaccard dissimilarity between genomes based on presence/absence information for each family. Hierarchical clustering was performed using the apcluster package.

Finally, pathway analysis was performed using the Protein Family Sorter tool from the PATRIC service. PATRIC was also used to scan for virulence genes.

### Data Deposition

Raw sequence data and assembled genomes were deposited in NCBI with human reads removed under BioProject accession number PRJNA312222.

## Results

We assembled a total of 90 genomic bins from the sequence data obtained from the four stool samples, and from these bins we identified 59 essentially complete genomes (see Materials and Methods for a description of how completeness was calculated). These genomes, including 24 previously announced by Jeraldo et al. (2015) but not yet described, came from diverse genera. Some genomes represented genera known to dominate the gut, including *Bacteroides* (*n* = 2), *Clostridium* (*n* = 3), *Eubacterium* (*n* = 2), and *Ruminococcus* (*n* = 3). *Lactobacillus* (*n* = 2), a genus known for its health promoting effects, was also represented. Other, less-common genera included *Acinetobacter* (*n* = 1); *Akkermansia* (*n* = 1), some species that are associated with healthier metabolic status (Dao et al., 2016); bile-tolerant *Alistipes* (*n* = 1); *Burkholderia* (*n* = 1); *Carnobacterium* (*n* = 2); *Odoribacter* (*n* = 3), increased levels of which, along with *Bacteroides*, have been linked to colon tumorigenesis in mice (Zackular et al., 2013); and *Roseburia* (*n* = 1), a genus enriched by dietary whole grains (Martínez et al., 2013). In addition, we identified a genome belonging to the Melainabacteria (*n* = 1), a proposed class in the Cyanobacteria (Soo et al., 2014) that is motile, non-photosynthetic, and capable of synthesizing several B and K vitamins (Di Rienzi et al., 2013).

### “Most Wanted” Species in Stool Metagenomic Data

We decided to focus on a single bin in more detail, and thus set out to identify the genomes of most interest. Fodor et al. (2012) composed a list of the “most wanted” taxa in the human microbiota, focusing on those that were prevalent in HMP volunteers and distantly related to already sequenced organisms. In the years that have elapsed, 1000s of new genome sequences have been collected, so we updated the “most wanted” list by using 16S data to investigate which bacterial taxa were most common amongst the gut samples from the MWRP (representative of the population from which our stool samples were collected) and which had matches in the GOLD database, which tracks current and past sequencing projects (**Figure 1A**). As expected, with the additional genomes in the public repository, the average similarity to sequenced genomes increased from 91.4% in Fodor’s HMP sample to 91.9% in our MWRP sample. Based on Fodor’s criteria (prevalence ≥20% and similarity to sequenced organisms ≤90%), we identified 13 “most wanted” complete and partially complete genome bins (see **Supplementary Table S1**).

From the genome bins deemed essentially and partially complete, we are focusing on a single bin with a 16S rRNA sequence closely matching an OTU from our updated “most wanted” taxa. The OTU was identified as being from the genus *Butyricicoccus*, with a prevalence of 97.5% in the MWRP and a sequence identity of 90.0% to the most related genome in the GOLD-Human and HMP databases. The genome bin was originally constructed using supervised assembly with MetaHIT metagenomic species MGS-46, and re-assembly was then performed.

This bin contained three genomes, one each from samples N15, N54, and K4410, and we began by investigating their taxonomical niche. Phylogenies based on both 16S RNA, good for resolving taxa at the species level and above, and whole-genome tetranucleotide spectrum data, good for resolving taxa below the species level, confirmed that the three genomes were most closely related to the species *Butyricicoccus pullicaecorum* and *Eubacterium desmolans* (**Figure 1B**). The ANIb among the three genomes varied from 94.4 to 96.3%, and they exhibited less than 76% similarity to the *B. pullicaecorum* and *E. desmolans* genomes (**Supplementary Figure S1**; **Supplementary Table S3**). Thus, these three genomes probably belong to the same species (the ANIb species cutoff is typically around 95%), a species that is quite distinct from *B. pullicaecorum* and *E. desmolans*. *B. pullicaecorum* is the sole known member of the genus *Butyricicoccus*, named for its ability to produce the anti-inflammatory SCFA butyrate. By contrast, *E*. *desmolans* belongs to a large and phylogenetically diverse genus that is the second most common in the human intestinal tract after *Bacteroides*. The *Eubacterium* genus as a whole is distinguished primarily by its negative metabolic characteristics (Schwiertz et al., 2000), but this particular species is known for its production of a steroid desmolase (Bokkenheuser et al., 1986). Because the assembled genomes’ closest known neighbors are both clostridial cluster IV/*C. leptum* subgroup species, we conclude that they belong to a novel clostridial cluster IV species. In keeping with the naming strategy proposed by Marakeby et al. (2014), which is based on genetic similarity, we designate these strains *Butyricicoccus* sp. B MC-N15, -N54, and -K4410. Here, the B indicates an ANI of approximately 70% from the nearest species, the MC indicates that the Mayo Clinic generated the sequences, and the final labels indicate the sample ID. From this point on, we will refer to the strains/genomes as N15, N54, and K4410 for simplicity.

### The N15, N54, and K44 Genomes

The K4410 and N54 genomes were each over 95% complete, whereas the N15 genome was roughly 80% complete (**Table 1**). The genomes encoded between 1,412 and 1,810 predicted genes, and GC content was roughly 55% for each.

**Table 1:** Characteristics of the three assembled *Butyricicoccus* sp. B MC genomes.

Genome	Assembled length (bp)	G + C%	Predicted genes	Estimated completeness (%)^a^
K4410	1877201	55.64	1,810	96.9
N15	1604539	55.01	1,543	80.8
N54	1452273	55.01	1,412	97.3


*^a^Calculated using Prodigal and HMMER version 3.0 (Eddy, 2011). We identified each bin’s essential genes using the hidden Markov models (HMM) used for essentiality tests in Albertsen et al. (2013), and we declared a bin “essentially complete” if we found 100 or more unique genes from this HMM set.*

**FIGURE 2:**
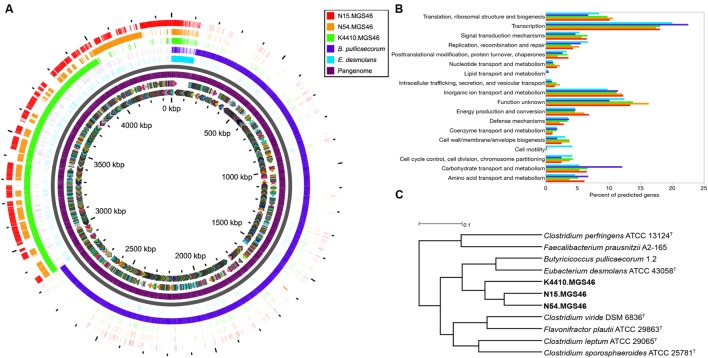
**(A)** A pangenome plot shows that the reconstructed N15, N54, and K4410 genomes display relatively high similarity to one another, except for a set of core genes; however, they differ substantially from the genomes of their closest known relatives, *Eubacterium desmolans* and *Butyricicoccus pullicaecorum*. From the inside to the outside of the circle: sense and antisense predicted genes, colored by cluster of orthologous groups (COG) category; “pangenome” track with predicted genes (*purple*); backbone (*gray*); *E. desmolans* predicted genes; *B. pullicaecorum* predicted genes; and *Butyricicoccus* sp. B MC N15, -N54, and -K4410 predicted genes, respectively, with genes shaded by identity to the pangenome predicted genes. **(B)** A COG usage bar chart shows that the N15, N54, and K4410 genomes have only a few potential motility genes, which encode the type IV pilus (in contrast to *E. desmolans*, which has many flagellar assembly genes), and they also possess few carbohydrate transport and metabolism genes (in contrast to *B. pullicaecorum*). No significant differences in gene category usage were apparent amongst the three reconstructed genomes. **(C)** FIGfams-based functional hierarchical clustering confirms that the nearest neighbors of N15, N54, and K4410 are *B. pullicaecorum and E. desmolans*. However, relationships to other organisms were different from those observed in the 16S or whole-genome phylogenies.

To learn more about the contents of the three assembled genomes, we performed a pangenome analysis in which N15, N54, and K44 were compared to one another and to the genomes of their closest known neighbors, *B. pullicaecorum* and *E. desmolans* (**Figure 2A**). The genomic content of the three strains was quite similar. By contrast, there was very little genomic overlap between these strains and their nearest neighbors. The pangenome analysis matches only genes with a BLAST identity of 80% or higher; the extensive white spaces in the comparisons of N15, N54, and K44 with *B. pullicaecorum* and *E. desmolans* underscore how distantly related this novel species is from currently sequenced organisms.

Next, we analyzed gene function. A COG usage bar chart illustrates the metabolic differences between N15, N54, K44, and their nearest neighbors (**Figure 2B**). Most notably, genes associated with cell motility are largely absent in the assembled genomes. They do contain genes for type IV pili, which allow gliding/twitching motion (**Table 2**), but unlike the *E. desmolans* genome, they do not possess genes for flagellar assembly. In addition, the number of genes associated with carbohydrate transport and metabolism is distinctly lower in the three assembled genomes than in *B. pullicaecorum*, which degrades carbohydrates. Amongst the N15, N54, K44 genomes, no significant differences in metabolic function stood out. When the novel strains were clustered with other clostridial cluster IV/*C. leptum* subgroup strains based on functional gene families, their two closest neighbors remained *B. pullicaecorum* and *E. desmolans*, despite substantial differences in gene content and function (**Figure 2C**). However, the relationships between the three novel genomes and more distantly related organisms were different from those observed in the 16S and whole-genome phylogenies; for example, *Clostridium sporosphaeroides* ATCC 25781 clustered quite near the three strains in the whole-genome phylogeny, but appeared not related in the functional analysis.

**Table 2:** Genes of interest in the three assembled *Butyricicoccus* sp. B MC genomes^a^.

Function	Gene or annotation	K4410	N15	N54
Sporulation	*spo0A*	Y	–	–
	*spo0J*	Y	–	–
	*spoIID*	Y	–	Y
	*spoIIP*	Y	Y	–
	*spoIIIAC*	–	Y	Y
	*spoIIID*	Y	–	–
	*spoIVA*	–	Y	Y
	*spoIVB*	Y	–	–
	*spoVAC*	–	Y	Y
	*spoVAD*	–	Y	Y
	*spoVAE*	–	Y	Y
	*spoVT*	Y	Y	–
	*yaaT*	Y	Y	Y
Cobalamin uptake	*btuCDF* complex	Y	Y	Y
Methionine uptake	*metNIQ* methionine ABC transporter complex	Y	Y	Y
Inositol uptake	Inositol transport system permease protein	Y		
	Inositol transport system sugar-binding protein		Y	
Thiazole uptake	*thiW*		Y	
Amino acid uptake	*artIJ*-like amino acid ABC transporter, amino acid-binding/permease protein	Y	Y	
Branched-chain amino acid uptake	*livFGHM*	Y		
Oligopeptide uptake	*oppA*	Y		Y
Sugar uptake	Sugar (glycoside-pentoside-hexuronide) transporter		Y	
Glycerol-3-phosphate uptake	Glycerol-3-phosphate ABC transporter	Y	Y	
Glutamate uptake	Glutamate transport ATP-binding protein		Y	
Polyol uptake	Various polyols ABC transporter	Y	Y	
Xyloside uptake	*xynT*	Y		
Hydroxymethylpyrimidine uptake	Hydroxymethylpyrimidine ABC transporter complex	Y		
Ammonium uptake	*amtB-glnK* Ammonium transporter		Y	
Type IV pilus	*pilB*	Y	–	Y
	*pilC*	Y	–	Y
Tetracycline resistance	*tetW*	–	–	Y
Antibiotic resistance	RND multidrug efflux transporter; acriflavin resistance protein	Y		Y
Antibiotic resistance	ABC-type multidrug transport system	Y		Y


*^a^Abbreviations: Y, yes (present); –, not present.*

**Table 2** lists a number of interesting genes identified in the N15, N54, and K4410 genomes. As in many of the Clostridia (Al-Hinai et al., 2015), a predominantly complete sporulation program, including the associated sigma factors, is present. In addition, a number of genes encode transporters that allow these strains to take up various nutrients from the environment. For examples, genes for cobalamin and methionine uptake are present in all three genomes, allowing this species to avoid the metabolically costly synthesis of these molecules. In addition, genes to take up branched-chain amino acids, oligopeptides, sugars, polyols, xyloside, glycerol-3-phosphate, and hydroxymethylpyrimidine are present. This diverse collection of transport genes suggests that these strains are scavengers dependent on other organisms for many essential molecules. Finally, several genes that potentially confer antibiotic resistance are present. A gene for tetracycline resistance, *tetW*, was detected in N54, and several efflux pumps were found in the reconstructed genomes as well. A scan using PATRIC failed to identify any virulence genes, however.

### Metabolism of N15, N54, and K4410

**FIGURE 3:**
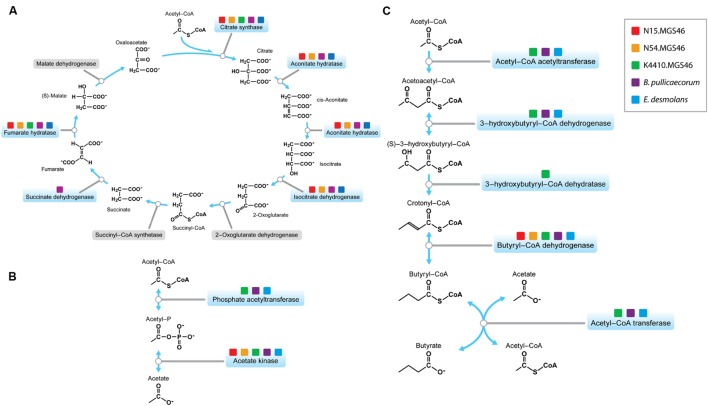
**(A)** The *Butyricicoccus* sp. B MC N15, -N54, and -K4410 genomes contain an incomplete tricarboxylic acid (TCA) cycle, a feature of anaerobes. The genes required for the TCA cycle that are present in these genomes are indicated by the colored boxes in the pathway map. **(B)** The N15, N54, and K4410 genomes support acetate production. The genes required for acetate production that are present in these genomes are indicated by the colored boxes in this pathway map; they indicate that acetate is produced from acetyl-P, mediated through an acetate phosphatase. **(C)** The N15, N54, and K4410 genomes support butyrate production, a trait shared by other members of the clostridial cluster IV/*Clostridium leptum* subgroup. The genes required for butyrate production that are present in these genomes are indicated by the colored boxes in this butyrate pathway map. The diagram shows a complete pathway for synthesis of butyrate, starting from acetyl-CoA exiting the TCA cycle.

Next, we took a more in-depth look at the metabolic genes in the three reconstructed genomes, to learn more about the potential niche of this bacterium in the human gut. We investigated whether the components of the tricarboxylic acid (TCA) cycle, a series of reactions essential for aerobic respiration, were intact. The cycle was incomplete (**Figure 3A**), with a pathway structure similar to that of *E. desmolans* and *B. pullicaecorum*. The genomes also lacked a gene for catalase (**Supplementary Table S2**), which protects aerobic organisms from oxidative damage. Thus, as expected from an intestinal member of the clostridia, these species are anaerobic.

Two of the main metabolic products of anaerobic fermentation by gut bacteria are the SCFAs acetate and butyrate, which modulate key processes in the gastrointestinal tract related to digestion and inflammation, and hence influence gastrointestinal health (Vinolo et al., 2011). Both of the strains’ nearest neighbors, *E. desmolans*, (Morris et al., 1986) and *B. pullicaecorum* (Geirnaert et al., 2014), produce acetate and butyrate. Therefore, we suspected that the three reconstructed genomes would encode the ability to produce these SCFAs as well. We first examined the potential of the N15, N54, and K4410 genomes to produce acetate. In this case, the gene for acetate kinase, the protein needed to convert acetyl-P to acetate, was present (**Figure 3B**), suggesting that the organism excretes the compound in the gut. Next, we determined whether the genomes support the production of butyrate. The five proteins necessary to synthesize this SCFA, starting from acetyl-CoA exiting the TCA cycle (**Figure 3C**), are acetyl-CoA acetyltransferase, 3-hydroxy-CoA dehydrogenase, enoyl-CoA hydratase, butyryl-CoA dehydrogenase, and acetate CoA-transferase; the genes for all of these proteins were present. This suggests that the organism is a butyrate producer, a trait shared by other members of the clostridial cluster IV/*C. leptum* subgroup.

## Discussion

Here we show that using readily available sequencing and analytic tools, we reconstructed the genomes of novel organisms from metagenomic data gathered from human stool samples. Current techniques allow us to zero in on organisms of interest, such as the “most wanted” taxa targeted in this paper, amidst sequences from thousands of other organisms. We exploited the following developments to reassemble essentially complete genomes from this complex sequencing environment: (1) We moved beyond paired-end sequencing to obtain longer, more reliable contigs, which make it easier for binning algorithms to separate organisms (Sharon et al., 2015; Kuleshov et al., 2016); and (2) We employed algorithms to quantify the completeness of candidate genomes based on conserved features (Parks et al., 2015), a strategy that works reasonably well even for novel organisms. By employing similar strategies, researchers should make rapid progress in sequencing and learning more about the HMP’s “most wanted” taxa and other uncultured organisms of interest to the research community.

The three complete genomes reconstructed here belong to a previously undescribed taxon. They are more than 94% similar to one another in terms of ANI; thus, it is appropriate to group them as representatives of a single species, one that we have designated *Butyricicoccus* sp. B MC.

Although 16S rRNA and whole-genome phylogenies place this species between *B. pullicaecorum* and *E. desmolans*, in the clostridial cluster IV/*C. leptum* subgroup of the Clostridiales, pangenome analysis indicates that the genomes of N15, N54, and K4410 differ substantially from those of their nearest neighbors in terms of both genes and functions. With a maximum ANI of 76.1% to other sequenced genomes, these strains are novel and unique additions to clostridial cluster IV.

Functional analysis of the N15, N54, and K4410 genomes suggests that this species is an anaerobe that occupies a scavenging niche in the gastrointestinal tract and produces butyrate. First, the species does not possess the molecules needed to complete the TCA cycle, and it does not encode catalase, an enzyme that protects aerobes from the toxic byproducts of oxygen metabolism. Second, the organism probably attaches to others cells in the gut. Genes for type IV pili/fimbriae are present; these sticky filaments are used to generate twitching motions, and they play an important role in attachment to and colonization of host cells (Mattick, 2002). Reliance on other cells, whether our own or those of other bacteria, is consistent with the lack of genes related to metabolic functions such as carbohydrate transport and metabolism. Third, despite this reliance, this species does not appear to be pathogenic: we found no evidence of virulence genes, and only several antibiotic resistance genes. Fourth, the species appears to produce the anti-inflammatory SCFAs acetate and butyrate, as it possesses complete or nearly complete pathways for both. Butyrate in particular is considered a “good” fatty acid, and researchers have been experimenting with the use of butyrate-producing bacteria to treat inflammatory bowel disease (Immerseel et al., 2010).

Of course, these are all genome-based predictions, and culturing these and other poorly understood organisms is an essential step in learning more about these bacteria, their metabolic and ecological niches, and their potential uses in medicine (Stewart, 2012). However, a genome sequence is a valuable tool when setting out to culture a novel bacterium. We observed that N15, N54, and K4410 are predicted to take up vitamin B12 (cobalamin) and methionine using the same transporter system. Uptake of the amino acid methionine is very common in bacteria; cobalamin is not only used as a cofactor for methionine biosynthesis, but all three genomes surveyed here also showed evidence for a B12-dependent radical SAM protein. This finding may provide a strategy for culturing *Butyricicoccus* sp. B MC: Supplementation with methionine and cobalamin may facilitate this secondary degrader’s growth. In addition, having a genome sequence on hand may enable researchers to synthesize probes useful for isolating the bacterium.

Our approach uses mate-pair sequencing to provide additional information for jumping large repeat gaps in the assembly process. While there are still repeats that cannot be resolved using this technique, using mate-pair libraries allows researchers to obtain scaffolds longer than previously possible. As an important limitation to note, this requires a significant amount of DNA and is limited to a subset of sequencing core providers. In addition, although mate-pair sequencing improves the quality of assembly, using longer contigs does not guarantee that contamination or misassembly will not occur. Thus, researchers will want to balance the quality of metagenomic assembly with the ability to sequence a larger number of samples, as the chance of obtaining multiple bins (albeit with shorter contigs) grows with the number of samples analyzed.

## Conclusion

We have obtained essentially complete genomes from stool metagenomic data using readily available methods, and that these genomes provide a rich source of culture-independent data about the “dark matter” of the human microbiome. Sequence data can be mined to generate a metabolic overview of an organism, which provides insight into its niche in the human body and what factors may be needed to culture it. Thus, the strategy presented here can be used to rapidly learn more about the HMP’s “most wanted” taxa, as well as poorly understood agents of disease. A concerted effort to sequence the genomes of these key players in the human microbiome is bound to lead to greater understanding of their roles in the gastrointestinal tract, and with luck, to information that can be used to maintain health and treat disease.

## Author Contributions

PJ and NC planned and designed the research; DA and LB collected samples; AH prepared the libraries and processed Illumina amplicon sequencing data; PJ, XC, and HN performed bioinformatic analyses; PJ, NG, BW, HN, and NC analyzed the data and wrote the paper; and all authors read and approved the final manuscript.

## Conflict of Interest Statement

The authors declare that the research was conducted in the absence of any commercial or financial relationships that could be construed as a potential conflict of interest.
