# Supplementary material for: Capturing One of the Human Gut Microbiome’s Most Wanted: Reconstructing the Genome of a Novel Butyrate-Producing, Clostridial Scavenger from Metagenomic Sequence Data
Source: Front Microbiol. 2016 May 26;7:783. doi: 10.3389/fmicb.2016.00783 (PMC4880562; doi:10.3389/fmicb.2016.00783)
Supplement: Supplementary file 4 [file Data_Sheet_1.DOCX]

Supplementary Material

Capturing one of the human gut microbiome’s most wanted: reconstructing the genome of a novel butyrate-producing, clostridial scavenger from metagenomic sequence data

Patricio Jeraldo, Alvaro Hernandez, Henrik Bjørn Nielsen, Xianfeng Chen, Bryan A. White, Nigel Goldenfeld, Heidi Nelson, David Alhquist, Lisa Boardman, Nicholas Chia^*^

*** Correspondence:** Prof. Nicholas Chia: chia.nicholas@mayo.edu

# Supplementary Figures and Tables

## Supplementary Figures

**Supplementary Figure 1.** **BLAST-based Average Nucleotide Identity (ANIb) phylogeny calculated for the reconstructed genomes and related organisms.** The three reconstructed genomes have high (>93%) ANIb, concordant with the 16S and tetranucleotide phylogenies. The other organisms have low (<70%) ANIb values, reaching the limit of reliability of the methodology.

## Supplementary Tables

**Supplementary Table 1.** Table of Operational Taxonomic Units (OTU) of the Midwest Reference Panel (MWRP) study with information about genome bins belonging to these OTUs. The table shows the OTUs’ prevalence in the MWRP dataset, the presence of metagenomic 16S reads matching the OTUs, the presence of reconstructed bins matching the OTUs, abundance of the OTUs expressed as a centered log ratio across all MWRP samples, the completeness level for the bins matching the OTUs, the total number of bins matching the OTUs (effective match count), the identities of the OTUs to the GOLD-Human database as calculated by USEARCH and VSEARCH, the designated priority for the OTUs based on their prevalence and identity to the GOLD-Human database, and the predicted taxonomy assignment of the OTUs using the SILVA database, version 123.

**Supplementary Table 2.** Complete coding sequence annotation of the three reconstructed genomes, as predicted by the PATRIC service, including predicted gene length, FIGfam ID (if available) and description of the product.

**Supplementary Table 3.** BLAST-based Average Nucleotide Identity (ANIb) distance matrix between the three reconstructed genomes and the genomes of related organisms. The table shows the high relatedness of the reconstructed genomes compared to the neighboring organisms of this clade. Given the two-way BLAST used to calculate ANIb values, the distance matrix is not symmetric.
